# Micro-Computed Tomography Analysis of Reciprocating Systems in Three-Dimensional Models of Mandibular Premolars with Two Canals

**DOI:** 10.3390/dj13040175

**Published:** 2025-04-19

**Authors:** María Medina-Gil, Ana Martín-Díaz, Natalia Navarrete, José Aranguren, P. S. Ortolani-Seltenerich, Giulia Malvicini, Gaya C. S. Vieira, Alejandro R. Pérez

**Affiliations:** 1 Department of Endodontics, Rey Juan Carlos University, Alcorcon, 28922 Madrid, Spain; mariamgventas98@gmail.com (M.M.-G.); anaamartindiaz@gmail.com (A.M.-D.); natnavarrete@hotmail.com (N.N.); josearanguren@hotmail.com (J.A.); 2 Department of Clinical Dentistry, Faculty of Biomedical and Health Sciences, Universidad Europea de Madrid, 28670 Madrid, Spain; 3 Department of Dental Pathology and Therapeutics, Faculty of Dentistry, UCAM, 30107 Murcia, Spain; psortolani@gmail.com; 4 Unit of Endodontics and Restorative Dentistry, Department of Medical Biotechnologies, University of Siena, 53100 Siena, Italy; giulia.malvicini@student.unisi.it; 5 Private Practice, Villa Nueva de Gaia, 4400-239 Porto, Portugal; dragayavieira@gmail.com

**Keywords:** endodontics, micro-CT, premolars, reciprocating systems, root canal preparation

## Abstract

**Objective:** This study investigated the shaping efficiency of four reciprocating systems—WaveOne Gold, Reciproc Blue, Excalibur, and One Reci—in three dimensional (3D) resin models of natural mandibular premolar teeth with two canals. **Methods:** Forty 3D-printed mandibular premolars (Vertucci configuration type V) were divided into four groups, each of which was assigned one of the reciprocating systems. According to the manufacturer’s protocols, each canal was prepared, with pre- and post-instrumentation micro computed tomography (micro-CT) scans evaluating canal volume, surface area, percentage of unprepared canal walls, and resin reduction in the pericervical area. Instrumentation time and screw-in sensation were recorded as qualitative performance indicators. Statistical analysis was performed using one-way ANOVA and chi-square tests with a significance of (*p* < 0.05). **Results:** All systems increased canal volume and surface area, primarily in the apical third, with Reciproc Blue and One Reci achieving the largest volume. WaveOne Gold had the highest percentage of unprepared walls (27.03%) and Reciproc Blue the lowest (19.65%), though these differences were not statistically significant (*p* > 0.05). Reciproc Blue caused the highest pericervical resin loss (22.24%), significantly higher than Excalibur (15.09%) and One Reci (15.17%) (*p* = 0.035). Reciproc Blue exhibited the highest incidence of screw-in sensation (70%), while WaveOne Gold achieved the shortest instrumentation time (86.7 s), although neither variable showed statistical significance. **Conclusions:** All systems effectively shaped complex canal anatomies, with Reciproc Blue demonstrating the highest dentin removal and WaveOne Gold proving the most time efficient. Clinically, these findings suggest that instrument selection should balance shaping efficiency with dentin preservation. Minimizing unprepared areas and preserving pericervical dentin are essential for enhancing disinfection and reducing the risk of root fractures, ultimately contributing to the long-term success of endodontic treatment.

## 1. Introduction

Mandibular premolars exhibit considerable anatomical variation within their root canal system, posing significant challenges for endodontic treatment. One notable variation is the presence of a lingual root canal, which is particularly common in mandibular first premolars. Studies show an incidence of 23.6% compared to 5.3% in second premolars [1,2]. According to Vertucci’s classification, type V is the most common configuration, followed by types I and III [1,2]. Understanding these anatomical variations is crucial, as incomplete instrumentation and disinfection of the root canal system are associated with persistent apical periodontitis [3,4]. A recent study has reported a 98% prevalence of post-treatment apical periodontitis in teeth with at least one untreated canal [3]. Additionally, the absence of adequate knowledge or failure to correctly manage such configurations can increase the risk of procedural errors, including canal transportation, ledging, and apical zipping. These complications may compromise the effectiveness of cleaning and shaping procedures and ultimately affect treatment outcomes [5].

Among the anatomical variations in mandibular premolars, Vertucci Type V is particularly relevant due to its clinical complexity. In this configuration, a single canal divides into two with separate apical exits, increasing the risk of missed anatomy and procedural errors, especially when the secondary canal is narrow, curved, or poorly visible on radiographs. The abrupt bifurcation and limited access to the lingual canal complicate detection and negotiation, often requiring advanced imaging or adjusted instrumentation. For these reasons, Type V anatomy serves as a clinically significant and standardized model for assessing the shaping efficiency, safety, and dentin preservation of modern endodontic systems [5,6].

Advancing imaging techniques have become essential, and cone beam computed tomography (CBCT) has been widely used to visualize this complexity because it produces high-resolution, three-dimensional images of the root canal system to aid diagnosis and treatment planning in complex cases [7]. However, micro-computed tomography (micro-CT) offers even higher resolution and allows a more detailed examination of internal tooth structures and unprepared canal walls [8]. This is critical, as studies have shown that 30–35% of mandibular premolar canal walls remain untouched by instrumentation [6,9], with 18–25% of these unprepared areas located in the apical third and potentially harboring microbial biofilms that compromise treatment success [6].

Alongside effective disinfection, the preservation of tooth tissue, especially pericervical dentin (approximately 4 mm above and below the crestal bone), is a critical factor in maintaining fracture resistance of the tooth under functional loads [10,11]. Recent studies emphasize that conventional access cavities may lead to excessive removal of healthy dentin, increasing susceptibility to fracture [10,11]. While minimally invasive approaches have been proposed to address this issue, their effectiveness remains a subject of debate [12,13].

Despite increasing interest in canal morphology and dentin preservation, only one study has examined the effectiveness of different instrumentation systems in shaping the lingual canals of mandibular premolars [14]. This is likely due to the difficulty of obtaining natural mandibular premolars with two canals. To date, no study has evaluated the removal of pericervical dentin in this group of teeth.

To overcome this limitation, reciprocating systems, such as WaveOne Gold (Dentsply Maillefer, Ballaigues, Switzerland), Reciproc Blue (VDW, Munich, Germany), Excalibur (Zarc4endo, Gijón, Asturias, Spain), and One Reci (Micro-Mega, Besançon, France), have been introduced to address the shortcomings of continuous rotary systems, particularly in reducing torsional loading and instrument fatigue [15]. With their unique thermal treatments and cross-sectional designs, these systems have been shown to achieve faster working lengths and reduce the risk of procedural errors [14,16].

Studies have shown the importance of selecting the appropriate nickel–titanium instruments [17,18]. Each system offers different advantages for maintaining the canal’s original shape while minimizing dentin removal [17,18].

Traditionally, such systems are tested using extracted natural teeth. However, anatomical variability among specimens [19] presents challenges in obtaining a sufficient number of comparable samples to evaluate multiple instrumentation systems within a single study. Additionally, the collection and storage of human teeth raise concerns about cross-infection risks [20] and pose difficulties in standardization.

As an alternative, plastic blocks with simulated canals have been suggested [21]. Still, these models are limited to single, simplified canal structures rather than replicating the intricate anatomy of whole teeth. Although commercially available models with realistic and standardized designs have emerged in recent years [22], these options are often expensive, have a restricted selection of tooth types, and exhibit variability in materials and production methods across brands [22].

3D printing technology, however, offers a promising solution for creating replicas that capture the complexities of natural tooth anatomy. This technology is gaining traction within dentistry [23] and, with an expanding range of printable materials, facilitates the production of tailored models applicable to various areas of endodontic research, such as irrigation, obturation [24], and chemomechanical preparation [25]. Studies utilizing 3D resin replicas [22] have found the printing accuracy sufficient for producing standardized tooth models beneficial in endodontic education.

Despite their advantages, printed teeth have limitations, particularly regarding material properties that differ from natural dentin [26]. Their hardness and radiopacity do not fully match those of human teeth [26]. Nonetheless, these models effectively simulate endodontic treatments [27] and present valuable opportunities for comparing various instrumentation systems [28].

In addition, this approach becomes valuable when obtaining complex anatomical samples suitable for evaluating shaping abilities and the remaining dentin is challenging [28]. It also allows us to standardize certain features, such as coronal access, in all cases and helps avoid introducing confounding variables into the study.

Therefore, this study compared the shaping effectiveness and the time efficiency of WaveOne Gold, Reciproc Blue, Excalibur, and One Reci in 3D-printed replicas of mandibular premolars with two canals (Vertucci Type V), focusing on their ability to preserve resin (simulating dentin) and navigate complex root canal anatomies.

## 2. Materials and Methods

### 2.1. The Sample Selection

No ethical approval was required for this study, as no human teeth, cells, or animals were used. The study was conducted with 3D-printed resin teeth, so an ethics committee review was not required.

Based on previous studies [28] and the expected differences between the systems, the aim was to identify meaningful differences with a statistical power of 80% and a significance level of 5%. Using G*Power 3.1 software (Heinrich Heine Universität, Düsseldorf, Germany), it was determined that 9 replicas per group would be sufficient to detect significant differences between the four groups. This resulted in 32 to 36 samples for the study, ensuring reliable and robust results.

Ten intact mandibular premolars with two canals and Vertucci classification V were selected and scanned using a micro-CT. To standardize the coronal access, 3D models of the teeth were created based on micro-CT scans. These scans were analyzed using 3D Slicer software (version 5.0.3) to generate stereolithography (STL) files. These files were then imported into Meshmixer (Autodesk Inc., San Francisco, CA, USA) to visualize and segment the root canals and dentin structures. A conservative access cavity was designed to minimize the removal of dentin and leave the pulp horns intact, and this access design was uniformly applied to all specimens.

### 2.2. 3D Resin Model Preparation

The process began with acquiring micro-CT scans of natural mandibular premolars with Vertucci type V canal configuration. These high-resolution scans were converted into STL files to serve as digital blueprints for 3D printing. The STL files were first imported into slicing software (AnyCubic PhotonWorkshop, Version 3.1.4) and edited to ensure optimal alignment and placement of the models on the print bed.

The models were printed using an Anycubic Photon Mono M5s printer (Anycubic Technology Co., Shenzhen, China), which utilizes stereolithography technology. The printer was loaded with UV-Tough resin (Anycubic Technology Co., Shenzhen, China), designed to operate within a UV wavelength range of 365–405 nm. To ensure accuracy, the printer operated at a resolution of up to 10 μm, enabling detailed anatomical replication.

This resin exhibits a density of 1.05–1.25 g/cm^3^ (Source: Anycubic) and a viscosity of 500–900 cP·mPa·s, making it suitable for fine detailing during printing. The material has a hardness rating of 71–75D (Source: Anycubic) and offers an elongation at break between 70 and 80%, providing both durability and flexibility in the printed models.

The resin’s flexural strength is rated between 15 and 25 MPa (Source: Anycubic), and its flexural modulus ranges from 350 to 800 MPa (Source: Anycubic), which supports structural stability. For thermal resilience, the resin has a heat deflection temperature of 50 to 55 °C, enhancing its suitability for applications where thermal tolerance is essential.

Although these values are lower than those of natural dentin, previous studies have demonstrated that such models adequately reproduce internal anatomy and allow for standardized assessments of shaping performance [22,23,25,28,29]. Their use eliminates inter-sample variability and provides a reproducible substrate for evaluating instrumentation effects, particularly in studies where ethical, anatomical, or logistical constraints limit the use of extracted human teeth.

Additional settings were adjusted to optimize exposure, with an initial exposure time of 20–40 s and a standard exposure time of 1.5–3 s per layer. The printed models have a storage life of approximately 1.5 years for long-term use, maintaining their properties and usability.

Each printing cycle took about 1.5 h. After printing, the models underwent a two-stage post-processing process. First, they were washed in 70% alcohol using an Anycubic Wash & Cure Plus device (Anycubic Technology Co., Shenzhen, China). This process removes uncured resin from the surface and internal structures, such as the canals.

After washing, the models were subjected to a 20 min UV curing process in the same machine. This curing step is essential for achieving the final hardness and ensuring that the fine anatomical features, such as the canal walls, remain intact and stable for further studies and instrumentation.

**Figure 1:**
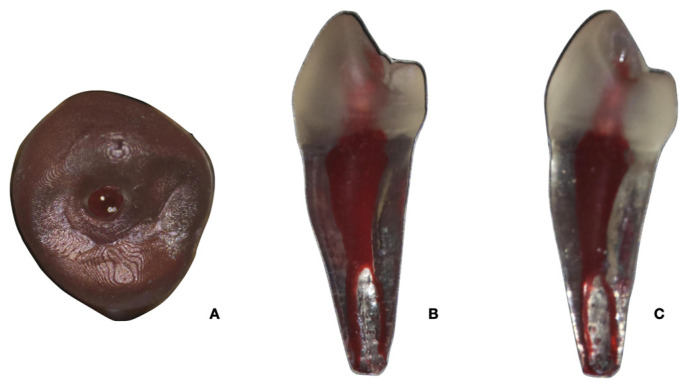
Photographs of the 3D-printed model from different views: occlusal (**A**), mesial (**B**), distal (**C**).

Finally, all models were carefully checked to ensure that they accurately replicated the original anatomy of the canal. Any inconsistencies were resolved by comparing the 3D models with the original micro-CT scans to provide an exact match with the natural tooth structures. This rigorous methodology ensured that the printed models were suitable for evaluating the shaping efficiency of the different NiTi systems investigated. Photographs of the 3D-printed model from different views are shown in Figure 1.

### 2.3. Chemomechanical Preparation of Root Canals

All 3D-printed teeth were first permeabilized with a K-type file No. 10 (Dentsply Tulsa Dental, Charlotte, NC, USA). The working length (WL) was established when the file was visible at the apical foramen. WL was set at 0.5 mm short of this length. The apex of each tooth was sealed with block-out resin (Ultradent, South Jordan, UT, USA), and the roots were embedded in condensation silicone (Zhermack, Badia Polesine, Italy) to stabilize the teeth during the experiment. This also created a closed system and simulated the vapor-lock effect.

Instrumentation of the root canal was performed by a single operator using a surgical microscope (Carl Zeiss, Oberkochen, Baden-Württemberg, Germany). The teeth were randomly assigned to one of four groups (*n* = 10 per group), each treated with four different instrumentation systems:

- WaveOne Gold Group (WOG): The WOG was used according to the manufacturer’s instructions. Instrumentation was performed with a ZEvo motor (Zarc4endo, Gijón, Spain) at 400 rpm, with a reciprocating motion of 170° counterclockwise and 50° clockwise. The WOG Glider (15/0.02) and Primary (25/0.07) files were used consecutively. At each 3–4 mm movement of the file, irrigation with 1 mL of 4.2% sodium hypochlorite (NaOCl) was performed with a Navitip 30G needle (Ultradent, South Jordan, UT, USA), followed by patency with a K-type file No. 10 at WL + 1 mm.
- Reciproc Blue Group: The Reciproc Blue System used an R25 (25/0.08) file in reciprocating motion, with 150° counterclockwise rotations and 30° clockwise rotations at 400 rpm with three strokes. After removing each file from the canal, it was rinsed with 1 mL of NaOCl (4.2%), followed by patency with a K-type file No. 10.
- Excalibur group: Instrumentation with the Excalibur system was performed with a ZEvo motor at 500 rpm with reciprocating movements of 150° counterclockwise and 30° clockwise with in and out movements. The system used E20 (20/0.05) and E25 (25/0.05) files, and irrigation with NaOCl (4.2%) was repeated at 3–4 mm intervals.
- One Reci group: The One Reci system used One Flare (17/0.09) and One G (14/0.03) files for glide path preparation, followed by One Reci (25/0.06). The files were operated at 400 rpm, with 170° counterclockwise rotations and 60° clockwise rotations, and irrigation was performed similarly to the other groups.

The total irrigation volume for all groups reached 10 mL of NaOCl. Before and after instrumentation was completed, all teeth were scanned with micro-CT.

To ensure procedural consistency, all instrumentation was performed by a single experienced operator who underwent dedicated training sessions on each of the four reciprocating systems prior to the experiment. This included multiple practice sessions on additional 3D-printed models with Vertucci Type V anatomy, ensuring familiarity with the systems’ working principles, motion kinematics, and instrument sequences as per manufacturer recommendations. Furthermore, to reduce the risk of operator fatigue and eliminate potential carryover effects, wash-out periods of approximately 15–20 min were observed between each instrumentation session.

### 2.4. Micro-CT Scanning

The 3D-printed teeth were scanned with a Phoenix V|tome|x S240 (General Electric, Boston, MA, USA). Scans were performed at 125 kV and 90 mA with an isotropic resolution of 20.0 μm. The teeth were rotated 360° during scanning, and a 0.2 mm aluminum filter was used to reduce beam hardening. The images were post-processed using Phoenix Datos|x 3D software, which generated approximately 1300 images per tooth. Artifacts were corrected with a 5% ring correction and a 50% beam hardening correction.

The surface area (mm^2^) and volume (mm^3^) of the root canal system were calculated before and after instrumentation, focusing on the full canal length (10 mm below the WL) and the apical third (4 mm below the WL). These measurements were performed using ImageJ software version 2.2 (National Institutes of Health, Bethesda, MD, USA). Unprepared areas of the canal walls were identified by subtracting the statistical voxels of the prepared canals from the pre-instrumentation and converting the results into percentage values. The 3D reconstructions were created with Meshmixer. Both canals were included for the evaluation, and the data were subsequently tabulated for the final comparison between the different systems.

To evaluate the pericervical resin (simulating dentin), the resin volumes before and after instrumentation were compared, with the volume of resin loss expressed as a percentage. The pericervical region was assessed at 8 mm from the coronal access of each tooth.

### 2.5. Screw-In Sensation and Instrumentation Time

The screw-in sensation was noted for each system during the procedure. The operator recorded whether this sensation occurred or not as part of the overall performance evaluation for each instrument system.

Instrumentation time was recorded from when the instrument was inserted into the canal until the last instrument reached the WL. The total time for each system was measured in seconds.

### 2.6. Statistical Analysis

The normality of the data was assessed using the Kolmogorov–Smirnov test, and homogeneity of variances was evaluated with Levene’s test. One-way ANOVA was applied to compare differences between the four groups for quantitative variables, including canal volume, surface area, percentage of unprepared canal walls, pericervical resin loss, and instrumentation time. When statistically significant differences were observed (*p* < 0.05), post hoc comparisons were performed using Tukey’s test. Categorical variables, such as the presence or absence of screw-in sensation, were analyzed using the chi-square test. All statistical analyses were performed using SPSS 21.0 (IBM, Armonk, NY, USA), and the level of significance was set at *p* < 0.05.

## 3. Results

### 3.1. Volume and Surface Area Changes

Table 1 shows the average values for volume and surface area in the total length of the canal (10 mm) and in the apical third (4 mm) of mandibular premolars before and after instrumentation with four different reciprocating systems: WaveOne Gold, Reciproc Blue, Excalibur, and One Reci.

**Figure 2:**
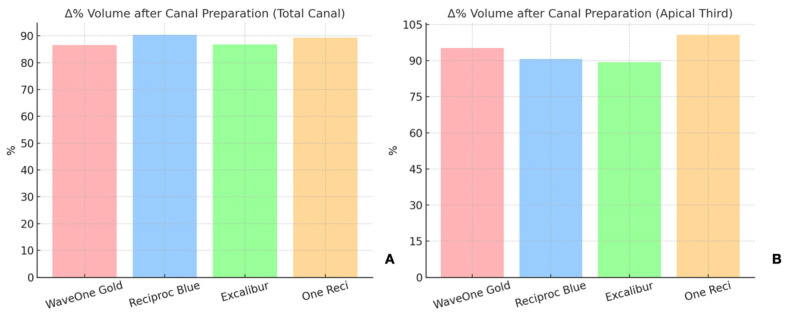
Relative increase in canal volume after preparation with the four reciprocating systems tested (WaveOne Gold, Reciproc Blue, Excalibur, One Reci). The graph on the left (**A**) shows the percentage increase over the full canal length (10 mm), while the graph on the right (**B**) focuses on the apical third (4 mm).

- In the total canal length (10 mm), the volume increased by 90.36% with Reciproc Blue, followed by 89.3% for One Reci, 86.75% for Excalibur, and 86.54% for WaveOne Gold (Figure 2A,B).
- In the apical third (4 mm), the volume increase was highest for One Reci at 100.67%, followed by 95.24% for WaveOne Gold, 90.64% for Reciproc Blue, and 89.41% for Excalibur.

**Figure 3:**
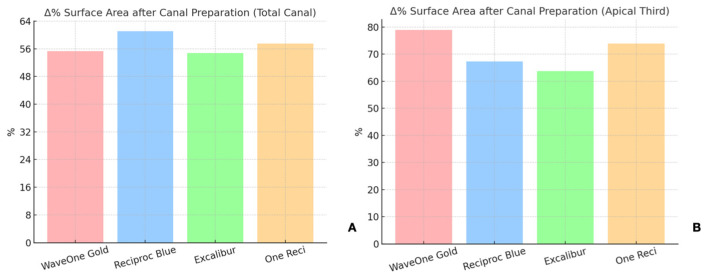
Relative increase in surface area after instrumentation in the (**A**) total canal and in the (**B**) apical third with the four reciprocating systems tested (WaveOne Gold, Reciproc Blue, Excalibur, One Reci).

The surface area also increased for all systems (Figure 3A,B).

- For the entire canal length (10 mm), WaveOne Gold increased the surface area by 55.36%, Excalibur by 54.78%, One Reci by 57.49%, and Reciproc Blue by 61.05%.
- In the apical third (4 mm), the increases amounted to 78.90% for WaveOne Gold, 73.93% for One Reci, 67.29% for Reciproc Blue and 63.71% for Excalibur.

Although differences in mean values were observed among the systems, no statistically significant differences were found between the systems regarding volume or surface area increase in the total canal length or the apical third (*p* > 0.05).

### 3.2. Unprepared Canal Walls

**Figure 4:**
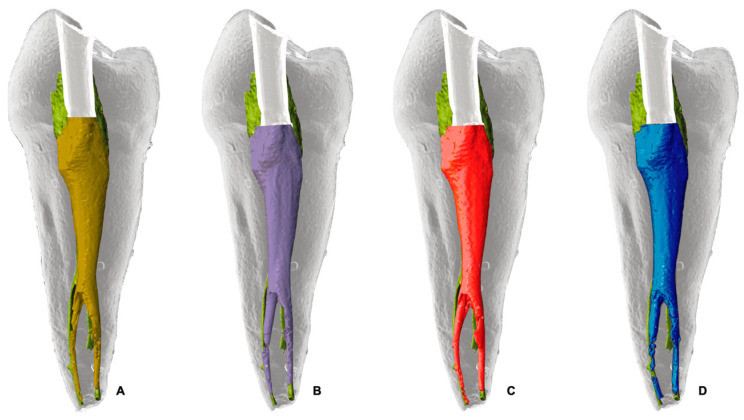
Schematic representation of unprepared canal walls (green) and instrumented areas in root canal preparations. (**A**) mustard yellow represents Excalibur; (**B**) purple, OneReci; (**C**) red, Wave One Gold; (**D**) blue, Reciproc Blue. Each color indicates the instrumented areas, highlighting differences in shaping outcomes by each system.

**Figure 5:**
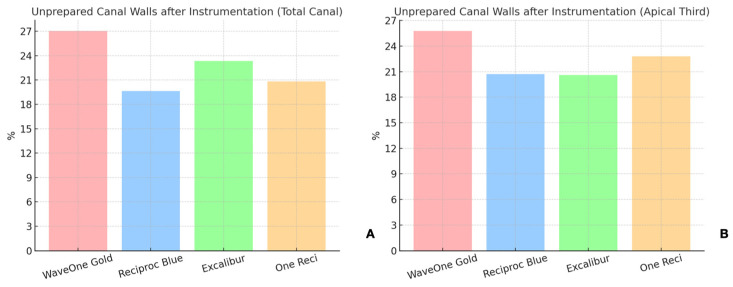
Unprepared canal walls after instrumentation with the four reciprocating systems tested (WaveOne Gold, Reciproc Blue, Excalibur, One Reci) in the (**A**) total canal and in the (**B**) apical third.

As shown in Table 1, the proportion of unprepared canal walls in the total canal length (10 mm) was highest for WaveOne Gold, at 27.03%, followed by Excalibur, at 23.33%, One Reci, at 20.85%, and Reciproc Blue, at 19.65% (Figure 4 and Figure 5). In the apical third (4 mm), the results were 25.76% for WaveOne Gold, 22.80% for One Reci, 20.70% for Reciproc Blue, and 20.59% for Excalibur (Supplementary Materials).

No statistically significant differences were found for the percentage of unprepared canal walls in the total length or the apical third (*p* > 0.05).

### 3.3. Pericervical Resin Conservation

**Figure 6:**
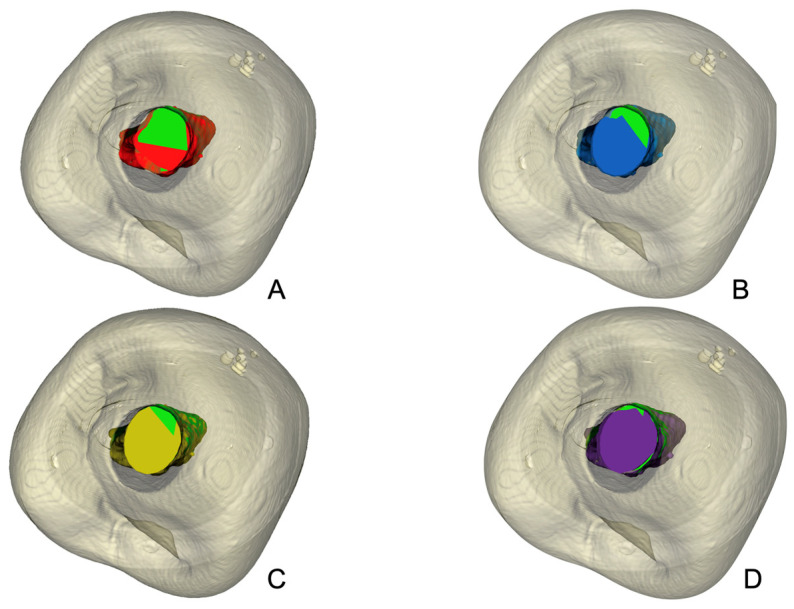
Illustrative outline of the pericervical resin (simulating dentin) during root canal preparation. Green represents the untreated canal, while dentin removal by each system is shown in color: (**A**) red for WaveOne Gold, (**B**) blue for Reciproc Blue, (**C**) mustard yellow for Excalibur, and (**D**) purple for OneReci. Each color emphasizes the dentin removal pattern of the respective system.

Reciproc Blue led to the highest reduction in pericervical resin (simulated dentin) with a decrease of 22.24%, followed by WaveOne Gold with 17.77%, Excalibur with 15.09% and One Reci with 15.17%. Significant differences were observed between Reciproc Blue and Excalibur and One Reci (*p* = 0.035), but not between Reciproc Blue and WaveOne Gold (Figure 6).

### 3.4. Screw-In Sensation

The operator reported the highest screw-in sensation for Reciproc Blue, which affected 70% of cases. It was followed by One Reci, with 50%, and Excalibur and WaveOne Gold, with 30% each. No statistically significant differences in screw-in sensation were found between the systems (*p* > 0.05). The results are shown in Table 2.

### 3.5. Instrumentation Time

One Reci required the longest time for canal preparation, an average of 201.40 s, followed by Reciproc Blue with 160.80 s, Excalibur with 129.50 s, and WaveOne Gold with 86.70 s. Although no significant differences in instrumentation time were found (*p* > 0.05), WaveOne Gold and Excalibur were more time-efficient than Reciproc Blue and One Reci.

## 4. Discussion

This study investigated the shaping efficacy of four reciprocating systems—WaveOne Gold, Reciproc Blue, Excalibur, and One Reci—in 3D-printed mandibular premolars with two canals, focusing on changes in canal volume and canal surface, unprepared canal walls, pericervical resin preservation, screw-in sensation, and instrumentation time. The results provide valuable insights into the strengths and limitations of each system and illustrate the complexity of endodontic treatment under challenging anatomies.

All systems significantly increased canal volume and surface area, with percentages ranging from 86.54% to 90.36%. This is consistent with previous studies [30,31] showing that reciprocating systems effectively enlarge canal spaces, particularly in the apical third, a critical area for microbial biofilm removal [32].

Increasing surface area up to 61.05% is crucial as it allows greater exposure to the irrigants, thus improving disinfection [33]. These results align with studies showing that flexible NiTi systems improve shaping in complex anatomies [14,34].

However, despite this significant volume and surface area increase, no significant differences were found between the systems. This suggests that while the effectiveness of shaping varies slightly between systems, the overall effects on root canal geometry do not differ significantly across systems, which is consistent with the results of other studies [9,35].

Unprepared canal walls are a critical challenge in endodontics [6,36], and the present study’s results confirm the previous literature on the persistence of this problem [32]. Some canal walls remained unprepared in all systems, with values ranging from 19.65% to 27.03%. This result aligns with other studies reporting similar figures for untreated surfaces after root canal preparation [6,37].

Untreated areas, especially in the apical third, pose a significant risk as they may harbor microbial biofilms and tissue remnants, which could jeopardize the treatment’s long-term success [4]. The current results are consistent with previous studies, which reported that despite using advanced systems designed to improve the canal system’s flexibility and adaptability, a higher percentage of prepared walls still cannot be achieved [32,36].

Importantly, these results highlight the effectiveness of modern reciprocating systems in preparing the canal while emphasizing the need for complementary cleaning methods, such as advanced irrigation techniques [38].

Although the differences observed among the tested systems did not reach statistical significance, a deeper analysis of the shaping patterns remains clinically meaningful. All instruments demonstrated the ability to effectively enlarge the root canal space and increase surface area, even in anatomically complex Vertucci Type V mandibular premolars [19]. This suggests that the selected systems are capable of negotiating and shaping these canal configurations while maintaining the original anatomy [39]. The observed differences in shaping behavior, though not statistically significant, may be attributed to intrinsic design characteristics, including cross-sectional profile, taper, alloy type, and thermal treatment [40]. For instance, the enhanced flexibility of heat-treated NiTi instruments (such as Reciproc Blue and WaveOne Gold) may contribute to improved centering ability and reduced canal transportation in curved or bifurcated canals [41]. Conversely, differences in cutting efficiency and debris removal could be influenced by variations in reciprocation angles and tip design [40,42].

Preservation of pericervical dentin is critical to maintaining tooth integrity after treatment [10]. While Reciproc Blue was effective in shaping, it resulted in greater resin loss, suggesting a more aggressive preparation approach. This may be attributed to its instrument geometry, which includes an S-shaped cross-section, a relatively high taper (25/0.08), and a larger core mass compared to other systems. These features contribute to greater cutting efficiency and debris removal but may also lead to increased dentin removal, particularly in the pericervical region [43,44]. This trade-off between effective shaping and dentin preservation has been well documented in different studies [12,45], highlighting the importance of balancing canal enlargement with structural preservation of the tooth.

As expected, the present findings showed that instruments with smaller taper profiles, such as One Reci (25/0.06) and Excalibur (25/0.05), preserved more pericervical dentin. Indeed, Reciproc Blue caused significantly more resin loss than One Reci and Excalibur (*p* = 0.035). However, WaveOne Gold (25/0.07), despite its greater apical taper, did not differ significantly. This may be due to its variable taper design, which decreases coronally and favors dentin preservation in the pericervical area. Additionally, differences in cross-sectional geometry and cutting efficiency likely influenced shaping behavior, reducing the expected differences based solely on taper. From a clinical point of view, systems with less taper, such as One Reci or Excalibur, which showed more conservative shaping, may be more suitable for cases where dentin preservation is of paramount importance [12].

The results emphasize the need to carefully consider the clinical implications of using more aggressive systems [46], particularly concerning their impact on long-term tooth survival. Excessive dentin removal in critical areas, such as the pericervical region, may increase the risk of root fractures [10], making system selection a crucial component of treatment planning [47]. This aspect is essential in mandibular premolars with complex anatomy, where excessive dentin removal may increase the risk of root fractures [11].

However, the results of this study should be interpreted with caution, as the resin used in the models is softer than natural dentin.

Systems with a higher taper could remove more resin in certain areas, a phenomenon that may not occur in natural teeth. However, the resin material used for fabricating the 3D-printed tooth replicas was selected for its mechanical properties, which approximate certain characteristics of human dentin. With a density of 1.05–1.25 g/cm^3^ and a flexural strength of 15–25 MPa (Source: Anycubic), the resin provides sufficient structural resistance for evaluating shaping performance, despite being softer and less rigid than natural root dentin, which exhibits a density of approximately 1.4–1.6 g/cm^3^ and flexural strength ranging from 200 to 270 MPa [48,49,50,51]. Although these properties make it a valuable standardized model for comparative studies, the results should be interpreted with caution, as instruments with greater taper may remove more resin than they would dentin under clinical conditions. Future studies using extracted human teeth with complex anatomies are needed to validate these findings and further assess the behavior of reciprocating systems in a biologically relevant environment.

Future studies should focus on comparing dentin removal in mandibular premolars with complex anatomy using different reciprocating systems.

A notable finding is the higher incidence of the screw-in sensation with Reciproc Blue compared to other systems. This result agrees with another study [52], conducted in severely curved canals, that observed operator control difficulties and the screw-in sensation [16]. Although that study was conducted in severely curved canals, an anatomical configuration different from the Vertucci Type V morphology, the findings are nonetheless consistent with the present study in highlighting the influence of instrument design on tactile feedback. This sensation can be problematic, especially in the apical third, where the risk of perforation is higher due to the thin dentin walls [53]. It is important to note that in this study, the Reciproc instrument was used as a single file, whereas the other systems involved the creation of a glide path using files with a smaller diameter and taper. Using a smaller diameter and taper before instrumentation with larger files, such as the No. 25, could reduce the likelihood of a “screw-in” sensation [54].

The subjective nature of the screw-in sensation assessment represents one of the limitations of the present study. While this approach reflects real clinical conditions, it lacks the objectivity provided by more advanced experimental methods. Torque and apical force measurement devices have been used to quantify screw-in tendencies during root canal shaping [55], while finite element analysis (FEA) has been applied to simulate stress distribution in instruments and tooth structures under mechanical load [56]. Additionally, strain gauge systems have enabled real-time measurement of forces acting on the instrument during use [56]. Incorporating such objective methods in future studies would enhance the reliability of screw-in sensation evaluation to support or refine clinical observations.

Using 3D-printed teeth in endodontic research offers significant advantages, particularly in standardization and reproducibility [22]. These models enable the creation of highly standardized specimens that allow precise comparison between different instrumentation systems under controlled conditions [22,25]. Unlike natural teeth exhibiting anatomical variations, 3D-printed teeth eliminate such discrepancies and allow direct performance comparisons [22]. This standardization is particularly valuable for replicating complex root canal configurations and facilitates the study of system efficacy under challenging cases [57]. In addition, 3D-printed models address ethical and practical concerns associated with using extracted human teeth by providing a consistent and unlimited supply for research and education without donor variability or logistical difficulties [58].

Another significant advantage is the ability to customize complex anatomies with specific canal morphologies using micro-CT data [25]. This approach ensures a detailed analysis of the performance of different systems in scenarios that mimic real clinical challenges.

However, despite these advantages, 3D-printed teeth cannot perfectly replicate the physical properties of natural dentin [59]. While the resin used in these models is suitable for morphological replication, it does not mimic the hardness, elasticity, or mechanical behavior of dentin during instrumentation [59]. As a result, differences in cutting efficiency and debris removal may not fully translate to clinical performance [60].

In addition, the surface texture of 3D-printed canals may not have the microscopic irregularities found in natural dentin [61], affecting friction and tactile feedback during instrumentation. Natural teeth have different calcifications, tubule densities, and organic material, all of which affect the interaction of the instruments with the canal walls and irregularities that are absent in 3D models [60]. This can lead to differences in instrument wear and the perception of screw-in forces compared to clinical conditions.

Another limitation concerns the irrigation dynamics. The interaction between the irrigants and the canal surfaces in 3D-printed models may not correspond to the complex behavior observed in natural teeth, particularly regarding the penetration of NaOCl [62].

The clinical implication of these findings is that all tested systems can be considered reliable for managing complex premolar anatomies, provided that fundamental principles—such as glide path creation, appropriate irrigation, and three-dimensional obturation—are observed. Nevertheless, given the high variability in root canal morphology, particularly the presence of undetected lingual canals, advanced diagnostic tools such as CBCT should be considered to reduce the incidence of missed anatomy and associated treatment failures.

## 5. Conclusions

In conclusion, although all four reciprocating systems effectively shaped the canal space, none outperformed the others in all parameters. Each system balances shaping efficiency, resin (simulation resin) preservation, and operator control. The use of 3D-printed teeth in this study offered several advantages for standardizing experimental conditions and replicating complex root canal anatomies. However, limitations must also be considered when interpreting the results and their clinical relevance.

## Figures and Tables

**Table 1:** Average volume, surface area, instrumentation time, and pericervical resin reduction in mandibular premolars following preparation with four reciprocating systems. Values are shown as means (min–max).

Data	WaveOne Gold [Mean (Min–Max)]	Reciproc Blue [Mean (Min–Max)]	Excalibur [Mean (Min–Max)]	One Reci [Mean (Min–Max)]
Total length (10 mm)				
Volume (mm^3^)				
Initial	8.18 (2.99–16.79)	8.17 (2.99–16.79)	8.16 (2.99–16.79)	8.19 (16.79)
Final	14.11 (10.30–25.70)	14.40 (10.35–28.74)	14.06 (9.85–25.03)	14.31 (10.37–26.74)
Δ% after canal preparation (%)	86.54 (53.07–253.18)	90.36 (38.65–282.94)	86.75 (43.96–263.21)	89.3 (51.81–259.87)
Surface area (mm^2^)				
Initial	64.44 (37.77–129.02)	62.25 (37.37–129.02)	64.32 (37.37–129.02)	64.5 (37.37–129.02)
Final	94.63 (70.98–155.67)	97.65 (69.82–175.33)	94.48 (72.44–159.47)	96.15 (71.47–170.23)
Δ% after canal preparation (%)	55.36 (20.66–197.72)	61.05 (14.84–256.97)	54.78 (14.42–194.84)	57.49 (27.62–210.78)
Unprepared root canal walls (%)	27.03 (11.31–43.31)	19.65 (9.14–36.39)	23.33 (9.58–36.93)	20.85 (9.32–37.03)
Apical third (4 mm)				
Volume (mm^3^)				
Initial	1.02 (0.64–2.25)	1.06 (0.64–2.25)	1.04 (0.64–2.25)	1.05 (0.64–2.25)
Final	1.94 (1.05–3.99)	1.90 (0.84–4.02)	1.92 (1.29–3.89)	1.94 (1.19–3.69)
Δ% after canal preparation (%)	95.24 (42.71–184.71)	90.64 (31.25–210.59)	89.41 (24.04–138.82)	100.67 (23.96–195.29)
Surface area (mm^2^)				
Initial	19.65 (12.39–45.60)	19.75 (12.39–45.60)	19.43 (12.39–45.60)	19.32 (12.39–45.60)
Final	34.90 (19.28–85.60)	33.20 (14.39)	31.90 (19.50–78.60)	33.74 (18.51–82.80)
Δ% after canal preparation (%)	78.90 (25.59–152.17)	67.29 (16.14–184.47)	63.71 (13.80–103.46)	73.93 (18.12–157.17)
Unprepared root canal walls (%)	25.76 (10.12–50.95)	20.70 (7.80–40.43)	20.59 (10.15–42.95)	22.80 (10.64–50.58)
				
Instrumentation time (s)	86.70 (61–122)	160.80 (47–737)	129.50 (74–296)	201.40 (132–477)
				
Pericervical dentin (mm^3^)				
Initial	177.10 (118–93–200.20)	177.10 (118–93–200.20)	177.10 (118.93–200.20)	177.10 (118.93–200.20)
Final	145.26 (100.02–179.16)	138.52 (89.44–176.76)	150.42 (101.40–176.93)	150.19 (101.03–177.62)
Δ% After canal preparation	17.77 (8.05–33.91)	22.25 (9.28–38.97)	15.09 (9.20–20.32)	15.17 (8.84–23.85)

**Table 2:** Frequency of Screw-In Sensation During Instrumentation with Four Reciprocating Systems, Reported as Sample (%).

Data	WaveOne Gold	Reciproc Blue	Excalibur	One Reci
Screw-in				
Yes	3 (30%)	7 (70%)	3 (30%)	5 (50%)
No	7 (70%)	3 (30%	7 (70%)	5(50%)
Total	10 (100%)	10 (100%)	10 (100%)	10 (100%)

## Data Availability

The original contributions presented in this study are included in the article. Further inquiries can be directed to the corresponding author.

## Supplementary Materials

The following supporting information can be downloaded at: https://www.mdpi.com/article/10.3390/dj13040175/s1. Video showing unprepared canal walls (green) and instrumented areas in root canal preparations.
